# ANALYSIS OF DOUBLE BALLOON ENTEROSCOPY: INDICATIONS, FINDINGS, THERAPEUTIC AND COMPLICATIONS

**DOI:** 10.1590/0102-6720201700020002

**Published:** 2017

**Authors:** Flávio Heuta IVANO, Izabela Rodrigues VILLELA, Lívia Fouani de MIRANDA, Thaísa Sami NAKADOMARI

**Affiliations:** Serviço de Endoscopia, Hospital Sugisawa (Endoscopy Service, Sugisawa Hospital, Curitiba, PR, Brazil

**Keywords:** Double-balloon enteroscopy, Small bowel, endoscopy, Gastrointestinal hemorrhage, Abdominal pain.

## Abstract

**Background:**

The double balloon enteroscopy is an important method for the endoscopic approach of the small bowel that provides diagnosis and therapy of this segment’s disorders, with low complication rate.

**Aim:**

Analysis of patients undergoing double balloon enteroscopy. The specific objectives were to establish the indications for this method, evaluate the findings by the double balloon enteroscopy, analyze the therapeutic options and the complications of the procedure.

**Methods:**

It is a retrospective analysis of 65 patients who underwent double balloon enteroscopy.

**Results:**

Sixty-five procedures were performed in 50 patients, 63.1% were women and 36.9% were men. The mean age was 50.94 years. The main indication it was gastrointestinal bleeding, followed by abdominal pain and Crohn’s disease. Most procedures were considered normal. Polyps were the most prevalent finding, followed by angioectasias and duodenitis. In 49.2% of the cases, one or more therapeutic procedures were performed, (biopsy was the most prevalent). There was only one case of acute pancreatitis, which was treated clinically.

**Conclusion:**

The enteroscopy is good and safe method for the evaluation of the small bowel, and its main indications are gastrointestinal bleeding and abdominal pain. It has low complications rates and reduces the necessity of surgery.

## RESUMO

**Racional:**

A enteroscopia por duplo balão é importante método endoscópico para abordagem do intestino delgado, permitindo o diagnóstico e a terapêutica de afecções com baixa taxa de complicações.

**Objetivo:**

Análise de pacientes submetidos à enteroscopia por duplo balão, estabelecendo as indicações para realização do exame e seus achados, analisando a terapêutica realizada e observando as complicações decorrentes do procedimento.

**Métodos:**

Estudo retrospectivo de análise de 65 prontuários de pacientes submetidos à enteroscopia por duplo balão. Os dados foram armazenados em planilhas e foi realizada a análise estatística.

**Resultados:**

Foram 65 enteroscopias em 50 pacientes, sendo 63,1% mulheres e 36,9% homens, com média de idade de 50,94 anos. A principal indicação foi sangramento intestinal, seguida por dor abdominal e doença de Crohn. A maioria dos procedimentos foi considerada normal, sendo que o achado mais prevalente foram os pólipos, seguido por angioectasias e duodenites. Em 49,2% dos exames foi realizado algum procedimento terapêutico, sendo a biópsia o mais prevalente. Houve apenas um caso de complicação de pancreatite aguda.

**Conclusão:**

A enteroscopia por duplo balão é bom e seguro método para a avaliação do intestino delgado, possuindo como principais indicações o sangramento intestinal e a dor abdominal. Possui baixas taxas de complicações e, por meio de suas opções terapêuticas, diminui a necessidade de procedimentos cirúrgicos.

## INTRODUCTION

The diagnosis of diseases involving the bowel is challenging due to the anatomy of this intestinal portion and the lack of tools for proper diagnosis. Recently they have been developed new methods that facilitated the approach of this site: the endoscope capsule and enteroscopy^5^.

The endoscope capsule allows reaching previously inaccessible areas, but does not allow the performance of biopsies or treatment of disease. In contrast, enteroscopy allows both diagnosis and therapy of disorders found. Although, this method avoids the need for several additional tests or intraoperative enteroscopies^2^ ^,^ ^5^.

There are three enteroscopy methods currently available: double balloon enteroscopy (DBE), single balloon enteroscopy and spiral enteroscopy. However, there are few studies that compare the different techniques and little is known about the benefits between the methods^5^ ^,^ ^10^.

Therefore, the main factors that determine the choice of method are the availability and endoscopist experience. In the present study, the technique available for analysis consisted in the DBE. It was developed in 2001 by Hironori Yamamoto, and it began to be used in 2004. It enabled the visualization of almost all the bowel, the collection of material for histologic evaluation and implementation of various therapeutic methods such as haemostasis, polypectomy and dilatation with balloon^2^ ^,^ ^5^.

DBE may be performed by anterograde or retrograde way, and the complete enteroscopy can be fulfilled by performing DBE by one extremity, marking the limit achieved with ink-of-china (nankin) and subsequent insertion of the enteroscope by the other extremity, until reaches the spot previously marked. It is known that the DBE has most complete enteroscopy realization rate compared too other methods^2^ ^,^ ^9^ ^,^ ^14^.

The indication of the insertion way varies according to the most likely location of the lesion, and, in the cases that the location is unknown; the choice is generally oral, since the retrograde technique is more complex and has higher rates of insucess^2^. It should be avoided in patients with altered anatomy by previous operations or disease, due to the risk of perforation. It is also not indicated for patients allergic to latex, since it is the balloon component. Currently, it is known that enteroscopy can be performed safely in children and adolescents^3^ ^,^ ^14^.

The double balloon enteroscopy is considered a safe exam, with low rates of major or minor complications. The most prevalent major complications are perforation, bleeding, acute pancreatitis and enteritis. Most commonly, there may be minor complications, which include abdominal discomfort and minimal trauma to the intestinal mucosa. It allows the patient to receive medical discharge in the same day^9^ ^,^ ^10^ ^,^ ^13^.

The aim of this study was the analysis of patients undergoing DBE establishing the indications, evaluation of findings, analysis of the therapeutic and complications from the procedure.

## METHODS

This study was conducted in accordance with the Ethical Guidelines established by the National Council of Health of the Ministry of Health. The research project was approved by the Research Ethics Committee (CEP) of the Pontifícia Universidade Católica do Paraná, under number 36476214.5.0000.0020. There was no interest’s conflict. It consisted in a retrospective analysis of medical records of all patients undergoing DBE in Sugisawa Hospital, Curitiba, Brazil, between 2011 and 2014. Were analyzed 65 electronic medical records and collected the following data: age, gender, indications for the procedure, findings and possible obtained diagnoses, therapeutic performed and further complications.

Inclusion criteria were patients undergoing DBE in the hospital and that had the report of the exam available for access. The exclusion criteria were the exams performed outside the period considered for the study.

Data were stored in Microsoft Excel spreadsheets program and then analyzed by the software SPSS version 20.0.

For references were analyzed publications of Medline/PubMed, Lilacs, Cochrane and SciELO since 2007. Searches were by descriptors: “double balloon enteroscopy”, “bowel,” “endoscopy”, “pancreatitis”, “gastrointestinal bleeding “ and “abdominal pain”. There were selected eleven articles to be used as basis for discussion.

## RESULTS

Overall, there were 65 double balloon enteroscopies in 50 patients. Of these, 63.1% were women and 36.9% were men. The age ranged between 19 and 91 years (mean 50.94, median 52±17.38 years).

### Indications

They were in prior bariatric and intestinal surgery, diarrhea, Crohn’s disease, abdominal pain, duodenal lymphoma, intestinal obstruction, gastrointestinal bleeding and Gardner’s syndrome, totaling nine indications. The most prevalent was the gastrointestinal bleeding, corresponding 35.4% of cases; the less prevalent was intestinal occlusion, duodenal lymphoma and diarrhea, with only one case each. It should be noted that among the 65 patients, 18 had no indication described in the electronic medical record, being considered as indeterminate indication (Table 1).

**TABLE 1:** Frequency of indications of double balloon enteroscopy

Indications	Frequency n (%)
Prior bariatric surgery	3 (4,6%)
Prior intestinal surgery	2 (3,1%)
Diarrhea	1 (1,5%)
Crohn´s disease	5 (7,7%)
Abdominal pain	6 (9,2%)
Duodenal lymphoma	1 (1,5%)
Intestinal oclusion	1 (1,5%)
Gatrointestinal bleeding	23 (35,4%)
Gardner´s Syndrome	5 (7,7%)
Indeterminate	18 (27,7%)
Total	65 (100%)

### Findings

The reports demonstrated 36 different findings, which were classified into 15 groups for analysis, namely: adenomas, angioectasias, angioectasias and pseudodiverticulum, diverticulum, duodenitis, stenosis, stenosis and atrophy, stenosis and pseudopolyps, elevated formations, gastritis, ileitis, ulcerated lesions, melaena, polyps and DBE without alteration (Table 2).

**TABLE 2:** Frequency of findings of double balloon enteroscopy

Findings	Frequency n (%)
Adenomas	1 (1,5%)
Angioectasias	7 (10,8%)
Angioectasia and pseudodivertículum	1 (1,5%)
Divertículum	2 (3,1%)
Duodenitis	4 (6,2%)
Stenosis	1 (1,5%)
Stenosis and atrofhy	1 (1,5%)
Stenosis and pseudopolyp	2 (3,1%)
Elevated formation	1 (1,5%)
Gastritis	1 (1,5%)
Ileitis	2 (3,1%)
Ulcerated lesions	3 (4,6%)
Melaena	1 (1,5%)
Polyps	9 (13,8%)
EDB without alterations	29 (44,6%)
Total	65 (100%)

Most exams (44.6%) were considered normal, with no visible alterations to enteroscope. The most prevalent finding was polyps, corresponding 13.8% of the exams, followed by angioectasias (10.8%). The third was most prevalent was duodenitis (6.2%), and the fourth was ulcerated lesions (4.6%). It was observed two cases of ileitis and two cases of diverticulum (Table 2).

The less found findings, and shown in only one exam each, were: adenomas, angioectasia associated pseudodiverticulum, elevated formations, gastritis of the excluded stomach and melaena (Table 2).

Four exams demonstrated stenosis, one in duodenum and three in the distal jejunum, wherein two of them associated to pseudopolyps and one to atrophy (Table 3).

**TABLE 3:** Findings of double balloon enteroscopy

Findings	Frequency (n)
Adenoma in duodenal papilla and ulcerated lesion in the jejunojejunal anastomosis	1
Duodenal and proximal jejunum angioectasia	1
Duodenal and proximal jejunum angioectasia; elevated formation in the proximal jejunum	1
Proximal jejunum angioectasias	1
Scattered angioectasias in duodenum and distal jejunum l	1
Meckel´s diverticulum	1
Colon diverticula and venous ectasias in the right colon	1
Duodenitis and extensive angiectasia in afferent loop	1
Erosive duodenitis, erosions and ulcers in the proximal and medium jejunum	1
Erosive duodenitis, middle jejunum mucosal thickening and exsudative erosions on distal jejunum	1
Infiltrative duodenitis	1
Duodenal stenosis	1
Distal jejunum angioectasia	1
Elevated formation in the terminal ileum	1
Ulcerated subepithelial elevated formations in the proximal jejunum and xanthomatous lesions in the distal jejunum	1
Ulcerated subepithelial formations in duodenum and lymphoid hyperplasia in the duodenal bulb	1
Angiectasia formations in the proximal jejunum and lipoma in the jejunum	1
Angiectases formations in the distal duodenum	1
Discret enanthematous gastritis of the excluded stomach (after bariatric surgery)	1
Internal hemorrhoids grade 2 and angiectases at rectosigmoid; pseudo diverticula of the sigmoid	1
Internal hemorrhoids grade 2 and mild erosive ileitis	1
Exudative ileitis	1
Ulcerated infiltrative lesions in duodenal with stenosis	1
Melaena	1
Normal	29
Polyp in middle jejunum	1
Polyp in the duodenum, proximal jejunum and middle jejunum	2
Polyp in proximal jejunum and ileum	1
Sessile polyps in the proximal jejunum	1
Duodenal polyps	1
Polyps in the duodenum and proximal jejunum	2
Jejunal polyp	1
Substenosis in middle jejunum and stenosis in distal jejunum with sentinel pseudopolyp	2
Substenosis on medium jejunum and distal jejunum stenosis; atrophy of the jejunal mucosa	1
Total	65

Findings with the highest average age were the angioectasia, in which was found a media of 68.1 years. Yet the diagnostics with lower media (34 years) were the stenosis associated with atrophy or pseudopolyp.

### Therapy

Of the 36 exams in which alterations were found in the DBE, 31 underwent some therapeutic method; one exam considered normal underwent biopsy and in the remaining interventions were not performed. In other words, in 49.2% of cases there was some therapeutic procedure during the realization of the DBE.

The most performed treatment was the excisional biopsy, totaling 18 procedures; one was associated with nankin tattoo spot**,** three to polypectomy, two to argon plasma coagulation (APC), among them one also associated with endoscopic mucosal resection (EMR) (Table 4).

The second most performed treatment was polypectomy (n=10), followed by APC (n=8). The relation of all procedures performed is described in Table 4.

**TABLE 4:** Frequency of performed therapy

Therapeutics	Frequency n (%)
Biopsy and polypectomy	3 (4,6%)
Biopsy	12 (18,5%)
Biopsy and tatoo with spot	1 (1,5%)
Sclerotherapy and APC	1 (1,5%)
APC	5 (7,7%)
APC and biopsy	1 (1,5%)
EMR and polypectomy	3 (4,6%)
EMR, APC and biopsy	1 (1,5%)
Polipectomy	4 (6,2%)
Tattoo with nankin spot	1 (1,5%)
None	33 (50.8%)
Total	65 (100%)

Among the nine exams diagnosed with polyps, only four were underwent to polypectomy exclusively. The remaining five went for one more type of therapy, such as biopsy, EMR and APC.

Of the seven exams with angioectasias finding, six underwent APC, wherein one associated with biopsy, and one was not subjected to any therapeutic procedure.

In the third most prevalent finding (duodenitis), biopsies were performed in three cases and APC associated with sclerotherapy with epinephrine in the remaining case. The nankin tattoo spot was performed in only two cases, one of diverticulitis and other of ulcerated lesion (Tables 5 and 6).

**TABLE 5:** Relationship between the findings and therapy - Part 1

	Therapeutics					
Findings	Biopsy and polypectomy n (%)	Biopsy n (%)	Biopsy and tattoo with nankin spot n (%)	Sclerotherapy and argon plasma fulguration n (%)	Argon plasma fulguration n (%)	Argon plasma fulguration and biopsy n (%)
Adenoma	0	1 (100%)	0	0	0	0
Angioectasias	0	0	0	0	5 (71,4%)	1 (14,3%)
Angioectasias and pseudodiverticula	0	0	0	0	0	0
Diverticula	0	0	1 (50%)	0	0	0
Duodenitis	0	3 (75%)	0	1 (25%)	0	0
Stenosis	0	1 (100%)	0	0	0	0
Stenosis and atrophy	0	1 (100%)	0	0	0	0
Stenosis and pseudopolyp	2 (100%)	0	0	0	0	0
Elevated formation	0	1 (100%)	0	0	0	0
Gastritis	0	1 (100%)	0	0	0	0
Ileitis	0	2 (100%)	0	0	0	0
Ulcerated lesions	0	1 (33,3%)	0	0	0	0
Melaena	0	0	0	0	0	0
Polyps	1 (11,1%)	0	0	0	0	0
DBE without alterations	0	1 (3,4%)	0	0	0	0
Total	3 (4,6%)	12 (18,5%)	1 (1,5%)	1 (1,5%)	5 (7,7%)	1 (1,5%)

**TABLE 6:** Relationship between the findings and therapy - Part 2

	Therapeutics				
Findings	Mucosectomy and polypectomy n (%)	Mucosectomy, argon plasma fulguration and biopsy n (%)	Polypectomy n (%)	Tattoo with spot n (%)	None n (%)
Adenoma	0	0	0	0	0
Angioectasias	0	0	0	0	1
Angioectasias and pseudodiverticula	0	0	0	0	1 (100%)
Diverticula	0	0	0	0	1 (50%)
Duodenitis	0	0	0	0	0
Stenosis	0	0	0	0	0
Stenosis and atrophy	0	0	0	0	0
Stenosis and pseudopolyp	0	0	0	0	0
Elevated formation	0	0	0	0	0
Gastritis	0	0	0	0	0
Ileitis	0	0	0	0	0
Ulcerated lesions	0	0	0	1 (33%)	1 (33%)
Melaena	0	0	0	0	1 (100%)
Polyps	3 (33,3%)	1 (11,1%)	4 (44,4%)	0	0
DBE without alterations	0	0	0	0	28 (96,6%)
Total	3 (4,6%)	1 (1,5%)	4 (6,2%)	1 (1,5%)	22 (50,8%)
Melaena	0	0	0	0	1 (100%)
Polyps	3 (33,3%)	1 (11,1%)	4 (44,4%)	0	0
DBE without alterations	0	0	0	0	28 (96,6%)
Total	3 (4,6%)	1 (1,5%)	4 (6,2%)	1 (1,5%)	22 (50,8%)

### Complications

There was only one case of complications due to enteroscopy evolving with acute pancreatitis after the procedure, representing 1.5% of the exams, which received clinical treatment with solution of the condition.

## DISCUSSION

The DBE is recent endoscopic technique which revolutionized the approach to bowel affections, since it is an effective method both for the diagnosis of diseases of this segment, and for the treatment of these findings during the examination. This possibility of treatment reduces the need for more invasive interventions and, consequently, operatory risks^1^ ^,^ ^3^ ^,^ ^11^ ^,^ ^13^ ^,^ ^16^.

### Indications and findings

Indications for EDB are multiple and are increasingly expanding because the procedure allows, besides the diagnosis of diseases, interventions like biopsies and other therapeutics^8^.

Gurkan et al. analyzed in Turkey the patients undergoing DBE between the years 2009 and 2011 and observed that the most common indication was abdominal pain, followed by diarrhea and bleeding. These data contrast with those obtained in the present analysis, in which the most prevalent indication was gastrointestinal bleeding, comprehending 35.4% of cases. Abdominal pain corresponded to the second indication, totaling 9.2%^3^. One hypothesis for the divergence of the findings between the two studies is that Gurkan et al. analyzed also the pediatric population and the study involved only 35 procedures. The analysis considered the largest number of DBE also found as the main indication the intestinal bleeding^3^.

In a study of 216 DBE, Pata et al. revealed the obscure gastrointestinal bleeding as the primary indication, corresponding to 42.1% of cases, according to the data obtained in this study^5^.

Jeon and Kim also exposed the obscure intestinal bleeding as the primary indication and, diverging from this article and Gurkan et al., the authors found abdominal pain as the third most prevalent indication^3^ ^,^ ^5^.

In a retrospective study between 2007 and 2011, Prachayakul et al. analyzed 145 enteroscopies and observed that the main indication for its realization was also the intestinal bleeding of undetermined origin, followed by chronic diarrhea^10^.

Semrad in 2009, also affirmed that the obscure intestinal bleeding is the primary indication for the enteroscopy. The author also mentions that Crohn’s disease, polypoid syndrome, chronic diarrhea and refractory celiac disease also correspond to important indications of enteroscopy. In this study, however, no DBE was performed due to celiac disease and there were five cases (7.7% of total) in which the indication of the DBE was Crohn’s disease, the third most prevalent indication^12^.

Xin et al. analyzed 12823 procedures and observed that the greatest indication was also the intestinal bleeding (presumed from bowel), corresponding to 62.5% of executed exams, data that coincides with the findings of He et al. (2003-2011) in 59 patients; 36 performed enteroscopies for intestinal bleeding, 15 for abdominal pain and three by diarrhea^4^.

Jeon and Kim observed that the most common DBE findings were ulcers and irregular mucosa, followed by erosions. In the present study, the most common findings were polyps, followed by angioectasias, and found only three cases diagnosed with ulcerated lesions (4.6%) and one elevated formation, which corresponds to irregular mucosa. The same authors remark that angioectasias corresponded to the fourth most common finding in his analysis^5^.

According to Ferro et al, in the literature are described some cases of Meckel’s diverticulum diagnosed by enteroscopy, which could not be found by other complementary methods. In the present study, there was only one case^2^.

He et al. also obtained different findings in their study, which found that the main enteroscopic findings were primary or metastatic tumors, followed by diverticula, ulcers and angioectasias. It should take into account that the high prevalence of tumors in the series of these authors was influenced by the mean age of patients - 69.63 years - unlike this article, which had age average of 50.94 years^4^.

### Therapy and complications

Therapeutic options of DBE are diverse and comprehend any procedure performed during enteroscopy with diagnostic and curative purpose, including biopsy, polypectomy, argon plasma coagulation (APC) and sclerotherapy with adrenaline injection^1^ ^,^ ^3^.

Gurkan et al. described the realization of polypectomy in patients with the diagnosis of jejunal polyps associated with biopsy, without further complications. In this study, the management in case of polyp was also polypectomy, and in five of the nine findings, was performed some other associated procedure, such as biopsy, mucosectomy or APC ^3^.

In cases of angiodysplasia, therapy described as the best choice is the APC, procedure also adopted in this service, since that in six of the seven findings of angioectasias, this was the chosen procedure^3^.

Another therapeutic option described in the literature was adrenaline injection to treat ulcers. In the reports analyzed for this study, sclerotherapy with epinephrine was used in one case of duodenitis with extensive angioectasia. Chen et al. reported the existence of one case described in the literature that treatment with epinephrine injection to the small intestine wall lesions led to necrosis, a complication not found here^1^.

As the DBE is a new method of enteroscopy, risks are still being established. However, endoscopic technique is considered safe, with an acceptable safety profile, presenting complication rates of around 1.7% according to the literature. In the present study, it was described only one case of complication, representing 1.5%, which corroborates the existent data^6^ ^,^ ^7^.

It should be considered as a complication of endoscopy any adverse event occurring in the first 30 days after the examination and negatively change the patient’s health. In the case of DBE, the possible complications described include: bleeding, intestinal perforation, intestinal necrosis after injection of epinephrine, paralytic ileus, as well as adverse effects related to anesthesia, such as hypotension, desaturation or aspiration pneumonia. However, in 2006 it was described the first case of pancreatitis after DBE and since then, several articles have linked as a complication of enteroscopy, and currently the most common and serious complication of this procedure^6^ ^,^ ^7^ ^,^ ^14^.

It is noteworthy that the risk of complications is significantly higher after performing therapeutic procedures when compared to exclusively diagnostics exams. According to the literature, the complication rate in merely diagnostic endoscopy is low (0.4-0.8%) but increases to 3-4% if performing therapeutic procedures, reaching 10% in the case of high risk therapeutic, such as resection of large polyps. However, acute pancreatitis is an exception to this situation, reported in 0.3% of DBE and prevailing in diagnosis exams^6^.

Gurkan et al. analyzed 35 patients undergoing DBE, among which three developed abdominal pain and elevated amylase levels after the procedure and one patient had intestinal perforation after performing APC due to diffuse angioectasia, complications not found in the present study. On the other hand, He et al. reported only minor discomfort complaints, not recording severe complications such as perforation and pancreatitis^3^ ^,^ ^4^.

The literature reports increased risk of perforation in patients with anatomical alterations in patients with previous operations that are underwent to retrograde DBE. However, in this study, no one that had previous gastrointestinal operation presented this complication^11^.

The only complication in this study was a case of acute pancreatitis, agreeing with the data from other analysis that this is the most prevalent adverse event after the realization of DBE. Kopacova et al. defined the post-DBE pancreatitis as abdominal pain with worsening or beginning after the procedure, associated with the serum amylase level three times higher than the upper limit, during the first 24 h after the exam, which requires, at least, two more days of hospitalization than planned. Thus, abdominal pain and hyperamylasemia not necessarily mean pancreatitis and therefore many mild pancreatitis cases may not be diagnosed in patients undergoing outpatient DBE, underestimating the prevalence of this complication^6^.

By the year 2010, it has been described 51 cases of acute pancreatitis as a complication of DBE, one of these was fatal. There are several hypotheses about the mechanisms that induce increases in serum amylase, leading to pancreatitis, one of the main possibilities that repeated movements and compressions performed by enteroscope balloon may cause injury in the pancreatic sphincter^6^ ^,^ ^7^.

The studied articles demonstrate clear prevalence of acute pancreatitis in anterograde compared with retrograde. Therefore, it is recommended, for a more precise evaluation, that separately evaluate the development of this complication after each type of DBE, so that the prevalence of this condition after enteroscopy by the oral way would be greater, ranging approximately between 1.5-2%. However, in this study there was insufficient data in the records analyzed for this differentiation.

Finally, Lo and Simpson recommend that, as there is still no clear information on how to prevent the development of pancreatitis after the procedure, all patients undergoing oral DBE should be warned of this risk prior to the exam^7^.

## CONCLUSION

The double balloon enteroscopy is a good and safe method for the evaluation of the bowel, having as main indications intestinal bleeding and abdominal pain. It has low rates of complications and, through its therapeutic options, reduces the need for surgical procedures.
